# In vitro antineoplastic effects of auranofin in canine lymphoma cells

**DOI:** 10.1186/s12885-018-4450-2

**Published:** 2018-05-03

**Authors:** Hong Zhang, Barbara J. Rose, Alex A. Pyuen, Douglas H. Thamm

**Affiliations:** 1 Department of Veterinary Clinical Science, College of Veterinary Medicine, China Agricultural University, Beijing, 100193 China; 2 Flint Animal Cancer Center, College of Veterinary Medicine and Biomedical Sciences, Colorado State University, 300 West Drake Road, Fort Collins, CO 80523-1620 USA; 3 Cell and Molecular Biology Graduate Program, Colorado State University, Fort Collins, CO USA; 4 Comprehensive Cancer Center, University of Colorado, Aurora, CO USA; 5 Present Address: Department of Small Animal Medicine and Surgery, College of Veterinary Medicine, The University of Georgia, 2200 College Station Rd, Athens, GA 30602 USA

**Keywords:** Dog, Proliferation, Cancer, Apoptosis, Gold

## Abstract

**Background:**

The orally available gold complex auranofin (AF) has been used in humans, primarily as an antirheumatic/immunomodulatory agent. It has been safely administered to healthy dogs to establish pharmacokinetic parameters for oral administration, and has also been used as a treatment in some dogs with immune-mediated conditions. Multiple in vitro studies have recently suggested that AF may possess antineoplastic properties. Spontaneous canine lymphoma may be a very useful translational model for the study of human lymphoma, prompting the evaluation of AF in canine lymphoma cells.

**Methods:**

We investigated the antineoplastic activity of AF in 4 canine lymphoid tumor derived cell lines through measurements of proliferation, apoptosis, thioredoxin reductase (TrxR) activity and generation of reactive oxygen species (ROS), and detected the effects of AF when combined with conventional cytotoxic drugs using the Chou and Talalay method. We also evaluated the antiproliferative effects of AF in primary canine lymphoma cells using a bioreductive fluorometric assay.

**Results:**

At concentrations that appear clinically achievable in humans, AF demonstrated potent antiproliferative and proapoptotic effects in canine lymphoid tumor cell lines. TrxR inhibition and increased ROS production was observed following AF treatment. Moreover, a synergistic antiproliferative effect was observed when AF was combined with lomustine or doxorubicin.

**Conclusions:**

Auranofin appears to inhibit the growth and initiate apoptosis in canine lymphoma cells in vitro at clinically achievable concentrations. Therefore, this agent has the potential to have near-term benefit for the treatment of canine lymphoma, as well as a translational model for human lymphoma. Decreased TrxR activity and increasing ROS production may be useful biomarkers of drug exposure.

## Background

Non-Hodgkin lymphoma (NHL) is the fifth leading cause of human cancer death and is the second fastest growing cancer with regard to mortality in people [1]. Likewise, lymphoma is one of the most common neoplasms encountered in dogs, with an annual incidence up to 134 per 100,000 dogs [2, 3]. The incidence of canine lymphoma is increasing over time from 8.7% of all canine tumor in 1955 to 14.69% in 2008 [4]. Canine and human lymphoma are generally characterized by a high rate of initial remission following conventional CHOP (cyclophosphamide, doxorubicin, vincristine and prednisone) based therapies; however, 95% of dogs and 30% of humans will succumb to drug-resistant relapse [5–8]. To date, lymphoma is still a serious condition for which there are unmet medical needs both in humans and dogs. For this reason, it is essential to develop novel strategies to improve the outcome of patients suffering from aggressive or therapy-resistant lymphoma.

Concerns regarding the applicability of most rodent cancer models to human patients include immune status, lack of clonal heterogeneity, tumor location (orthotopic versus heterotopic), relative tumor burden, differences in drug distribution/metabolism, and differences in “achievable” drug concentrations. All of these contribute to the relatively well-documented poor correlation between results of many mouse studies and subsequent early clinical trials with anticancer agents [9]. More predictive animal models would, in many cases, appear warranted. The dog may be an extremely useful model for the study of lymphoma in humans, owing to striking similarities in histology, biology and gene expression. This includes identical gross, radiographic and histological appearance, similar locations of incidence and patterns of organ involvement, similar prognostic factors, and conserved dysregulation of signaling and growth regulation pathways [3, 10–14].

Gold compounds have been used in medicine dating as far back as 2500 BCE. Their primary use has been as immunomodulatory agents for the treatment of inflammatory diseases such as rheumatoid arthritis. The oral gold compound 2,3,4,6-tetra-o-acetyl-1-thio-β-D-glucopyrano- sato-S-(triethyl-phosphine) gold, manufactured under the drug name auranofin (AF, Ridaura®), was developed in the 1980s as a potential antirheumatic agent. More recently, antiproliferative and pro-apoptotic activity has been observed in a variety of human tumor-derived cell lines, including carcinomas of the breast [15–17], head and neck [18], ovary [19, 20], lung [21], and a variety of hematopoietic tumors [8, 22–24], including lymphoma [25]. Potential mechanisms of antineoplastic activity include modulation of secretion of tumor-promoting cytokines such as IL-6 and IL-8 by monocytes and macrophages [26–30], modulation of intracellular signaling and survival pathways such as MAPK, NF-kB, STAT3 and telomerase activity [15, 16, 22, 29, 30], and intracellular generation of reactive oxygen species (ROS) through inhibition of thiol-redox enzymes such as thioredoxin reductase (TrxR) and thioredoxin glutathione reductase [17, 18, 31, 32].

Several studies have evaluated the bioavailability, tolerability, pharmacokinetics and efficacy of AF in normal dogs and in dogs with immune-mediated disease. The oral bioavailability of AF ranges from 15 to 38% [33, 34]. To date, there is no information about the effect of AF in the treatment of canine lymphoid malignancies. In this study, we demonstrate that AF has potent antiproliferative and proapoptotic effects and synergy with lomustine (CCNU) or doxorubicin (DOX) in vitro against canine lymphoid tumor cells, suggesting the possibility of future studies evaluating this novel therapy as meaningful translational steps. Furthermore, this study has the potential to position dogs with spontaneous lymphoma as a viable model for future evaluations of AF therapy in human hematopoietic neoplasia.

## Methods

### Cell culture

The 1771 canine primitive/dedifferentiated B-cell lymphoma cell line was provided by Dr. K. A. Jeglum (Wistar Institute, Philadelphia, PA) [35]. The CLBL-1 canine B-cell lymphoma cell line was provided by Dr. B. C. Rütgen (Veterinary University of Austria) [36]. The OSW canine T-cell lymphoma cell line was provided by Dr. W. Kisseberth (The Ohio State University) [37]. The CLL-1390 canine primitive T-cell leukemia was provided by Dr. S. E. Suter (University of California, Davis) [38]. The human lymphoid tumor cell lines Raji and Daudi were obtained from American Type Culture Collection (Manassas, VA). All cell lines were maintained in C/10 media [RPMI 1640 culture medium (Lonza, Walkersville, MD, USA) supplemented with 1× MEM vitamin solution (Cellgro, Henderson, VA), 2 mM L-glutamine (Cellgro), 1 mM sodium pyruvate (Cellgro), 1 × non-essential amino acid solution (Cellgro), 1 × antibiotic/antimycotic (Cellgro) and 10% heat inactivated fetal bovine serum (FBS) (Peak, Fort Collins, CO)] in a humidified incubator, with 5% CO_2_ at 37 °C. All cell lines were confirmed to be of canine origin by multispecies multiplex polymerase chain reaction (PCR) and authenticated by short tandem repeat analysis as described [39].

### Growth inhibition assay

Cell lines were plated in 96-well plates in C/10 medium at a cell density of 1× 10^4^ per well, to which serial dilutions of a 100 mM DMSO stock solution of AF (Sigma, St. Louis, MO) were added in quintuplicate. Cells were then incubated for 72 h at 37 °C. Relative viable cell number was determined using a bioreductive fluorometric assay (Alamar Blue, Promega, Madison, WI) according to manufacturer directions, using a Synergy HT plate reader (Bio-Tek, Winooski, VT) and expressed as a percentage of control-treated cells. Each experiment was repeated at least three times and mean [± error (SE)] calculated. The 50% inhibitory concentration (IC50) for growth inhibition was calculated by fitting to a sigmoidal dose-response curve using Prism 5.0 (GraphPad Software, La Jolla, CA).

### Annexin V/propidium iodide assay

Apoptosis was measured in CLBL-1 and CLL-1390 cells using the V450 Annexin V and propidium iodide (PI) (BD Biosciences, CA), following the manufacturer’s instructions. Briefly, at 24 or 48 h after treatment with 250 nM or 1 μM AF, cells were harvested and washed twice with ice-cold PBS, and then resuspended in 1 × binding buffer. Annexin V and PI were added and analyzed by flow cytometry (CyAN ADP flow cytometer, Beckman Coulter, Indianapolis, IN) within 1 h. Unstained cells in buffer were assessed to evaluate auto-fluorescence. Treated cells were stained individually with Annexin V and PI to define population boundaries. Untreated cells were stained with both V450 Annexin V and PI to establish the basal level of apoptosis and necrosis. The percentages of early (Annexin V-positive, PI-negative) and late (Annexin V-positive, PI-positive) apoptotic cells were quantified using Summit Software 4.3 (Beckman Coulter).

### Thioredoxin reductase enzymatic activity

TrxR activity was assessed using a commercially available enzymatic assay (Cayman Chemical, Ann Arbor, MI), which is based on the reduction of DTNB (5,5′-dithio-*bis* (2-dinitrobenzoic acid)) with NADPH to 5-thio-2-nitrobenzoic acid (TNB). Briefly, cells were treated with various concentrations of AF, then collected by centrifugation at 1000–2000×g for 10 min at 4 °C. The cell pellet was homogenized in 0.5–1 mL of cold buffer (50 mM potassium phosphate, pH 7.4, containing 1 mM EDTA), and centrifuged at 10,000×g for 15 min at 4 °C. The supernatant was removed and stored on ice. The samples were then added to 96-well plates in the presence and absence of an included TrxR inhibitor (ATM). Diluted assay buffer in the presence and absence of ATM was added as background. Rat liver TrxR was used as a positive control. All the samples and controls were assayed in duplicate. The reactions were initiated by adding 20 μL of NADPH and 20 μL of DTNB to all wells. The microtiter plate was carefully shaken for 10 s to mix. The absorbance was read once every 1.5 min at 405–414 nm using a BioTek plate reader. Each experiment was repeated three times and mean [± standard deviation (SD)] calculated. The following formulas were used to determine the change in absorbance (∆A_405_) per minute, corrected ∆A_405_ per minute and to calculate TrxR activity.

$$ \Delta {A}_{405}/\mathit{\min}.=\frac{A_{405}\left( Time\ 2\right)-{A}_{405}\left( Time\ 1\right)}{Time\ 2\left(\mathit{\min}.\right)- Time1\ \left(\mathit{\min}.\right)} $$

$$ Corrected\ \Delta A/\mathit{\min}.(sample)=\Delta A/\mathit{\min}(sample)-\left[\Delta A/\mathit{\min}\left( sample+ ATM\right)-\Delta A/\mathit{\min}\left( Bkg+ ATM\right)\right] $$

$$ TrxR\ Activity\left(\mu mol/\mathit{\min}/ mL\right)=\frac{Corrected\ \Delta A/\mathit{\min}.(sample)\ }{7.92{mM}^{-1}}\times \frac{0.2\  mL}{0.02\  mL}\times Sample\ Dilution $$

(Note: Bkg = Background)

### ROS assay

The cell-permeable dye 2′,7′-dichlorodihydrofluorescein diacetate (H_2_DCFDA) (dichlorofluorescin diacetate) is a chemically reduced form of fluorescein that is used as an indicator of ROS activity in cells. CM-H_2_DCFDA is a chloromethyl derivative of H_2_DCFDA, which exhibits better retention in live cells than H_2_DCFDA. The lymphoma cells were harvested and resuspended in PBS with 10% FBS at a concentration of 1 × 10^6^ cells/mL. All test conditions were in quintuplicate. The cells were treated with CM-H_2_DCFDA (Life Technologies, Carlsbad, CA) at a concentration of 5 μM for 30–45 min at 37 °C in the dark. After incubation, cells were washed once with PBS and resuspended in PBS with 10% FBS, then cells were treated with various concentrations of AF. Unstained cells with CM-H_2_DCFDA were used as a negative control. Cells treated with t-butylhydroperoxide (tBHP) (Sigma-Aldrich, Saint Louis, MO) at final concentration of 1 mM were used as positive controls. The plates were read every 30 min for the first 2 h on a BioTek plate reader at 485/535 nm, then every hour until equilibrium was reached or the values started to drop. The data for all conditions were corrected by subtracting the reading of the unstained wells. Each experiment was repeated three times and mean [± SD] calculated. To interrogate the effect of ROS on cell growth, the canine lymphoma cells were treated with AF with or without 50 μg/mL trolox, a potent free radical scavenger. After 72 h co-incubation, relative viable cell number was detected using the bioreductive fluorometric assay described previously. Each experiment was repeated three times and mean [± SE] calculated.

### Combination of AF with DOX and CCNU

The antiproliferative activity of AF combined with DOX and CCNU, which are common cytotoxic drugs used in canine lymphoma treatment, was evaluated by the constant ratio combination design proposed by Chou and Talalay [40]. Tumor cells were seeded at a density of 1 × 10^4^ cells per well in 96-well plates, and supplemented with various concentrations of AF, DOX, CCNU and combinations of AF with DOX or CCNU. The constant combination ratio experiment was carried out at an equipotency ratio [i.e., (IC50) drug 1/(IC50) drug 2]. The concentration ratio of AF and DOX in combination was 4:1, 12:1, 4:1, and 3:1 in 1771, CLBL-1, OSW and CLL-1390 cell lines, respectively. In the combination of AF and CCNU, the ratio was 1:2000,1:3000, 1:200 and 1:500 for 1771, CLBL-1, OSW and CLL-1390 cell lines, respectively. Cells were incubated for 72 h at 37 °C. Relative viable cell number was determined using Alamar Blue as described above. To calculate the combined drug effects, the combination index (CI) and the dose-reduction index (DRI) were generated using CompuSyn software (ComboSyn, Paramus, NJ). CI values 1, < 1, and > 1 indicated an additive effect, synergism or antagonism, respectively. To further evaluate the combined effect, 1771 and OSW cells were treated with various dilutions of AF, DOX, CCNU, AF + DOX, and AF + CCNU. After 72 h incubation, relative viable cell number was determined as above. Each experiment was repeated three times and mean [± SD] calculated.

### The effect of AF on primary canine lymphoma cells

Primary canine lymphoma samples were collected from the Colorado State University Veterinary Teaching Hospital (CSU-VTH) through surgical excision of a lymph node, performed according to CSU-VTH standard operating procedures and with informed owner consent and approval of the CSU Institutional Animal Care and Use Committee. The samples were dissected and minced into 2–3 pea-sized lymph node pieces and 2–3 mL sterile PBS with Penicillin/Streptomycin (PBS/PS) added. The tissues were gently mashed with the end of a 12 mL syringe until homogenized. Those cells were separated through a 70 μm and 40 μm cell strainer (BD Biosciences, CA, USA) into a 50 mL centrifuge tube. The cell suspension was underlaid with 2 mL lymphocyte separation media (LSM, Mediatech, Manassas, VA) and centrifuged at room temperature for 18 min at 2200 RPM. The lymphocyte band was removed at the gradient surface, then resuspended with PBS/PS and centrifuged for 10 min at 1000 RPM. The cell pellet was re-suspended, counted and seeded into T75 flasks with 12 mL C/10 RPMI 1640 medium supplemented with 50 ng/mL recombinant tetrameric human CD40 ligand (megaCD40L, Enzo Life Sciences, Farmingdale, NY). When the cells grew well, cell growth inhibition was assessed according the protocol described previously.

### Statistical analysis

Statistical analysis was performed using Prism 5.0. The statistical significance of differences among multiple comparisons were performed by analysis of variance followed by one-way ANOVA on ranks with Tukey’s post hoc test. Statistical tests with *p* < 0.05 were considered significant.

## Results

### Auranofin has potent antiproliferative and proapoptotic effects in vitro

**Fig. 1:**
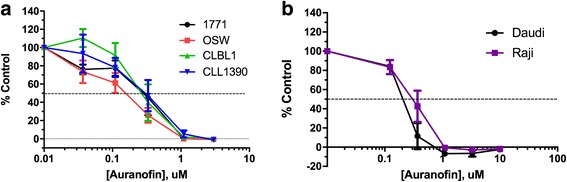
Auranofin exhibits dose-dependent growth inhibitory effects in canine and human lymphoid tumor cells. 4 canine (**a**) and 2 human (**b**) lymphoid tumor cell lines were incubated with AF for 72 h, followed by determination of relative viable cell number using a bioreductive fluorometric assay. Curves represent means of three independent experiments, and error bars indicate SEM

**Fig. 2:**
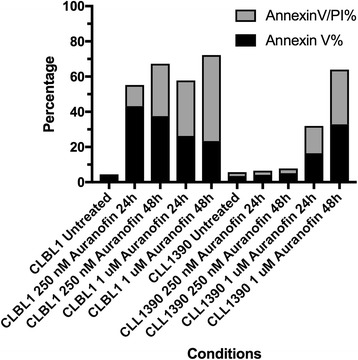
Auranofin enhances apoptosis of canine lymphoma cells in a dose- and time-dependent manner. CLBL-1 and CLL-1390 cells were incubated with 250 nM or 1 μM AF for 24 or 48 h, then stained with Annexin V and PI and analyzed by flow cytometry. The cells stained with Annexin V-positive and PI-negative were in early stage of apoptosis and those stained with Annexin V-positive and PI-positive were in late apoptosis or dead

To assess the antiproliferative effects of AF in canine lymphoid tumor cell lines, we performed growth inhibition assays using a bioreductive fluorometric assay. Auranofin demonstrated dose-dependent antiproliferative effects in all 4 cell lines, with IC50 values ranging from 0.16 to 0.33 μM (Fig. 1a). This is less than the 0.7–1 μM mean plasma steady-state concentrations achievable in humans with chronic dosing [41], and similar to those we observed in human lymphoma cells (Fig. 1b). Since the bioreductive method used above cannot distinguish between cell growth inhibition and cell death, Annexin V/PI double staining was utilized to quantify the apoptotic population by flow cytometry. As shown in Fig. 2, AF increased total apoptosis ratios in both early (Annexin V-positive, PI-negative) and late (Annexin V-positive, PI-positive) apoptotic cells in a dose- and time-dependent fashion. Taken together, these results showed AF induced cell growth inhibition and apoptosis in canine lymphoid tumor cell lines.

### Auranofin decreases thioredoxin reductase activity

**Fig. 3:**
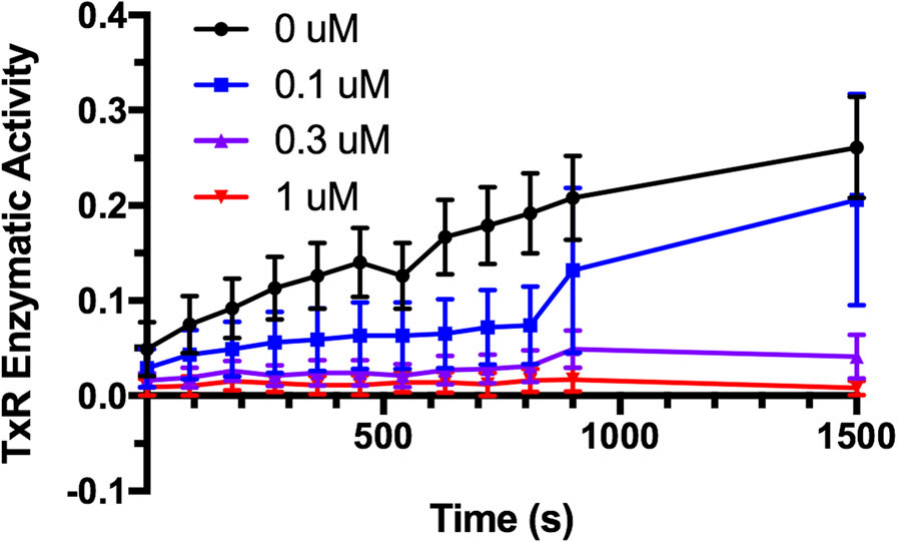
Auranofin suppresses canine lymphoma thioredoxin reductase enzymatic activity. The OSW T cell lymphoma cell line was incubated with AF at various concentrations, followed by serial determination of TrxR activity using a commercial enzymatic assay. Curves represent means of triplicate values, and error bars indicate SD

Thioredoxin reductase is known to be the only physiological reductase to reduce Trx, which has a wide range of functions in cellular signaling [42, 43]. To explore potential mechanisms of action of AF and identify putative pharmacodynamic (PD) markers for drug exposure, we assayed TrxR activity in canine lymphoid tumor cells after the treatment with AF. Treatment with AF led to dose- and time-dependent inhibition of TrxR activity in canine lymphoid tumor cells (Fig. 3).

### Auranofin enhances reactive oxygen species production

**Fig. 4:**
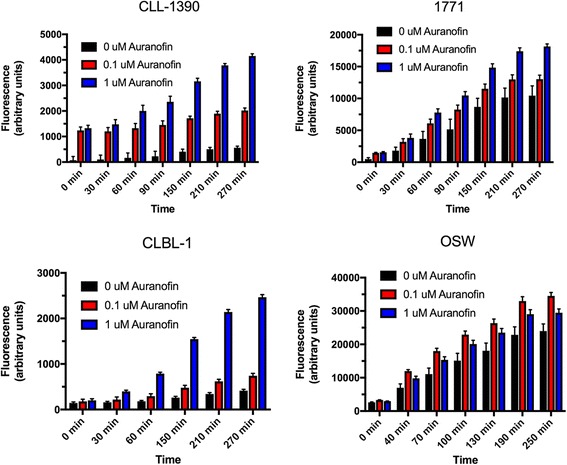
Auranofin induces reactive oxygen species formation. Four cell lines were incubated with varying concentrations of AF, followed by serial determination of ROS levels using the redox-sensitive fluorophore CM-H2DCFDA. Bars indicate means of triplicate measures and error bars indicate SD

Reactive oxygen species (ROS) is a collective term which is composed of superoxide anion (O_2_-), hydrogen peroxide (H_2_O_2_) and hydroxyl radical (-OH). Augmented intracellular ROS has been demonstrated to be associated with cell cycle arrest, senescence, and cancer cell death [44]. To further investigate the mechanism of AF-induced cell death and apoptosis, we employed CM-H_2_DCFDA, a fluorescent ROS-sensing probe, to evaluate the generation of ROS in the canine lymphoid tumor cells following the treatment with AF. As shown in Fig. 4, treatment with AF induced ROS accumulation in all 4 canine lymphoid tumor cells in a time- and dose-dependent manner. However, 2 of the cell lines (1771 and OSW) demonstrated time-dependent increases in ROS in the absence of AF. While AF enhanced ROS production, the differences were not statistically significant in these 2 cell lines. Interestingly, co-incubation with the potent free-radical scavenger trolox did not protect cells from AF-associated growth inhibition (not shown), suggesting that increased ROS generation might not be the primary antitumor mechanism of AF in these cells.

### AF synergizes with DOX and CCNU

**Fig. 5:**
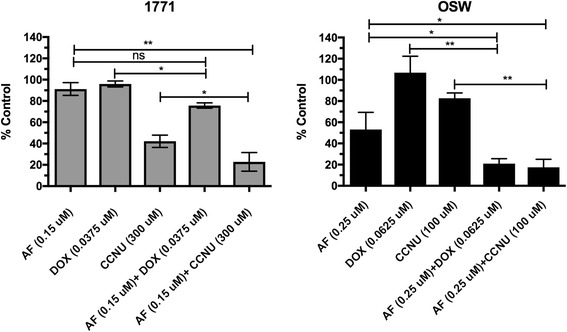
Auranofin synergizes with DOX and CCNU in canine lymphoma cells. Canine lymphoma cell lines 1771 and OSW were incubated with AF, DOX, CCNU, AF + DOX or AF+ CCNU for 72 h, followed by determination of relative viable cell number using a bioreductive fluorometric assay. Bars represent means of three independent experiments, and error bars indicate SD. The significance of differences between groups was analyzed by one-way ANOVA on ranks with Tukey’s post hoc test. ** *p* < 0.01, * *p* < 0.05

To evaluate the potential to combine AF with standard therapies, we employed the Chou and Talalay method to evaluate combinations of AF with other commonly used cytotoxic drugs for canine lymphoma treatment. The combination effects of AF, DOX and CCNU in canine lymphoid tumor cell lines are summarized in Table 1, as represented by CI (combination index), DRI, which is a measure of reduced fold of each drug dose in a synergistic combination at a given effect level, and the dose-effect levels of cell growth inhibition (ED50-ED95). The AF/CCNU combinations were synergistic at all dose levels in the 1771 and CLL-1390 cell lines (CI < 1), and synergistic effects were also exhibited in OSW and CLBL-1 cells, except at high dose levels, i.e. ED95. Interestingly, synergistic effects were noticed at all dose levels for the combination AF/DOX in OSW; however, the AF/DOX combinations were additive or antagonistic at all dose levels in 1771, CLBL-1 and CLL-1390 cells. The DRI represented a considerable dose reduction for all drugs in the combination due to their synergism. For instance, when employing synergistic drug combinations at the responding dose levels, the DRI showed that the concentration of AF and CCNU to inhibit 95% cell growth could be decreased 4.16-fold and 2.14-fold in 1771 cells, respectively, when AF was combined with CCNU. The dose of DOX was decreased 3.12-fold to cause 50% cell growth inhibition when AF combined with DOX in OSW. Moreover, the relative viable cell number was significantly different by comparing single drugs with combination treatments (*p* < 0.05) in 1771 and OSW, with the exception the combination of AF and DOX in 1771 cell line (shown in Fig. 5), which further confirmed this synergistic effect. These results suggest that combined treatment with AF and conventional cytotoxic drugs such as DOX and CCNU could result in enhanced antitumor activity*.*

**Table 1:** Dose-effect relationships of single drugs and combinations in canine lymphoid cell lines

Cell line	Single drugs and combinations	Parameters			CI value at				DRI value at			
		Dm	m	r	ED50	ED75	ED90	ED95	ED50	ED75	ED90	ED95
1771	AF	0.10	−2.27	− 0.97								
	DOX	0.07	−1.19	− 0.96								
	CCNU	161.99	−1.71	−0.98								
	AF + DOX	0.14	−2.37	−0.99	1.44	1.68	2.03	2.38	0.93	0.91	0.89	0.87
									3.00	1.74	1.10	0.81
	AF + CCNU	73.70	−1.73	−0.88	0.81	0.77	0.73	0.71	2.80	3.24	3.76	4.16
									2.20	2.18	2.16	2.14
OSW	AF	0.34	−2.09	−0.98								
	DOX	0.09	−1.69	−0.96								
	CCNU	229.43	−0.76	−100								
	AF + DOX	0.15	−2.29	−0.97	0.67	0.74	0.83	0.90	2.89	2.75	2.62	2.54
									3.12	2.63	2.21	1.97
	AF + CCNU	18.12	−1.96	−0.95	0.35	0.45	0.71	1.08	3.74	3.87	4.00	4.09
									12.72	6.44	2.17	1.19
CLBL-1	AF	0.15	−2.22	−0.98								
	DOX	0.05	−1.92	−0.98								
	CCNU	118.01	−1.25	−0.93								
	AF + DOX	0.14	−2.27	−0.97	1.03	1.06	1.08	1.10	1.02	1.18	1.17	1.16
									5.17	4.74	4.35	4.35
	AF + CCNU	64.88	−1.64	−0.9	0.69	0.80	0.93	1.05	6.96	8.28	9.85	11.08
									1.82	1.48	1.20	1.04
CLL-1390	AF	0.62	−1.83	−0.92								
	DOX	0.34	−2.28	−0.91								
	CCNU	235.94	−1.96	−0.97								
	AF + DOX	0.66	−2.67	−0.99	1.27	1.47	1.71	1.89	1.26	1.04	0.86	0.75
									2.10	1.95	1.82	1.73
	AF + CCNU	86.74	−1.87	−0.9	0.64	0.64	0.63	0.63	3.60	3.56	3.51	3.48
									2.73	2.80	2.88	2.94

*Dm* median- effect dose (concentration that inhibits cell growth by 50%), *m* shape of the dose-effect, *r* linear correlation coefficient of the median-effect plot

### Auranofin inhibits the growth of canine primary lymphoma cells

**Fig. 6:**
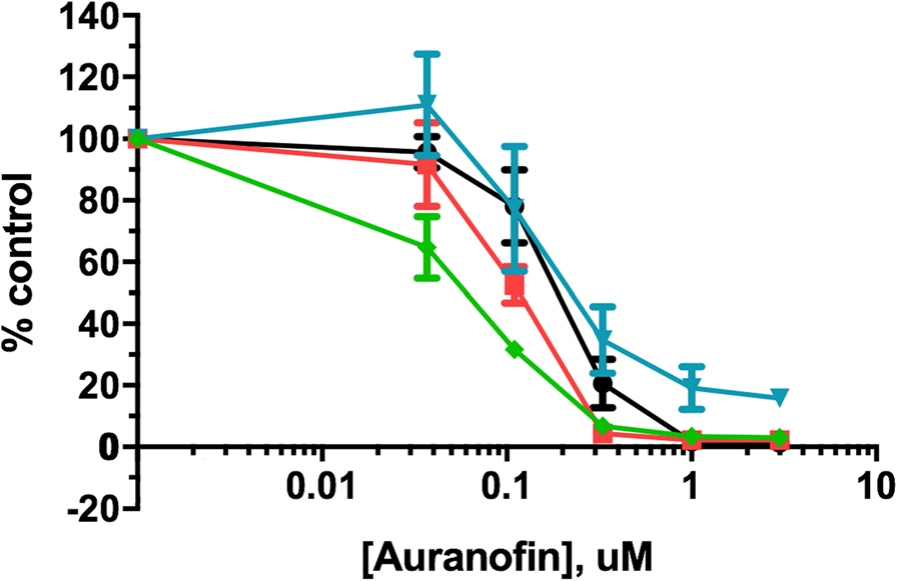
Auranofin inhibits the growth of canine primary lymphoma cells in a dose-dependent manner. Four canine primary B-cell lymphoma cultures were incubated with AF for 72 h, followed by determination of relative viable cell number using a bioreductive fluorometric assay. Individual colors represent cells derived from individual dogs. Curves represent means of three independent experiments, and error bars indicate SD

To detect the impact of AF in primary canine lymphoma cells, we collected tumor samples and carried out the growth inhibition assay described above for the lymphoid tumor cell lines. Four canine primary B-cell lymphoma samples were successfully grown in short-term culture. Three dogs were 6 years old, and one was 12 years old at the time of diagnosis. Represented breeds were Labrador retriever (2 cases), boxer (1 case) and coonhound (1 case). Auranofin attenuated the growth of canine primary lymphoma in dose-dependent manner, with IC50s in an equivalent range to that observed in the lymphoma cell lines (Fig. 6).

## Discussion

Multidrug chemotherapy protocols such as CHOP are the most effective and commonly used treatments for canine lymphoma, having been used for more than two decades [45]. The reported response rates can be greater than 85%, and survival times range from 8 to 12 months in most reports [6, 7, 46]. However, treatment of dogs with CHOP relapsed/refractory disease is considerably more challenging, and despite a panoply of investigated agents and protocols, response rates are lower and response durations shorter in these patients [47]. Hence, novel anti-lymphoma agents and protocols are needed to improve the initial outcome, extend remission durations, and provide additional treatment options for relapsed/refractory disease.

Auranofin is an agent that has approved by the US FDA for decades for the treatment of rheumatoid arthritis in humans, and has exhibited potential therapeutic activity for many other diseases, including neurodegenerative disorders, HIV/AIDS, parasitic/bacterial infections and cancer [48]. In this study, we have demonstrated that AF can inhibit the growth of canine lymphoid tumor cell lines and primary lymphoma cells, and trigger apoptosis in a dose- and time-dependent manner, at drug concentrations well within steady-state plasma levels achieved in humans. Based on this information, AF appears to be a reasonable candidate drug for the treatment of canine lymphoma.

The thioredoxin system consists of NADPH, Trx and TrxR, and is critical for the control of cell processes such as cell survival and apoptosis [42]. Cellular ROS homeostasis is regulated by stringently balancing of ROS-generating and scavenging systems such as superoxide dismutases (SOD1, SOD2, and SOD3), glutathione, catalase, and Trx [49]. A series of studies in lymphoma models and human lymphoma patients have demonstrated that attenuated expression of antioxidant enzymes and increased activity of the Trx system results in more aggressive cancer phenotypes and worse clinical outcomes [50–52]. Therefore, treatments directed at TrxR inhibition may be a potential therapeutic strategy for treatment of lymphoma and other cancers. In the light of the “hard and soft acids and bases” theory, gold complexes are likely to bind to the selenium atom in TrxR and thereby reduce the activity of TrxRs in both the cytosol and mitochondria [53]. Recent studies reveal that AF acts as an inhibitor of TrxR1, which is involved in oxidative damage and regulation of cellular redox signaling, followed by ROS excess and induction of apoptosis [20, 54]. In this study, we found that cellular responses to AF treatment were shifted from proliferation to cell death by attenuating TrxR activity (Fig. 3) and elevating intracellular ROS production (Fig. 4) in canine lymphoid tumor cells; however, co-incubation with the free radical scavenger trolox did not attenuate growth inhibition, suggesting that alternate mechanisms of action may be responsible for AF’s anticancer effect in these cells. Auranofin has been suggested to have multiple other potential mechanisms of action, including modulation of key cell signaling pathways, modulation of cytokine production, and inhibition of proteasomal activity, among others [15, 16, 22, 29, 30, 55]. Additional mechanistic studies are underway to identify potential mechanisms of AF’s effects in canine lymphoma cells. Nevertheless, TrxR activity and ROS production may also be useful as pharmacodynamic markers of drug exposure in future studies of AF in canine lymphoma.

A clinical trial of single-agent AF has been performed in human chronic lymphocytic leukemia (CLL) at the University of Kansas; however, preliminary studies suggest that enhanced efficacy is observed when AF is combined with other therapeutics [56]. Hence, evaluation of novel AF combinations will be dramatically accelerated with appropriate clinical evaluation in dogs with lymphoma. Many agents are used to treat canine lymphoma clinically, including DOX and CCNU. Our results demonstrated that the combination of AF with DOX or CCNU was synergistic and equivalent antitumor activity could be observed with reduced drug concentrations in certain canine lymphoid tumor cell lines. For example, as shown in Table 1 and Fig. 5, the concentration of CCNU could be markedly decreased to obtain similar inhibitory effects as a single-agent after combining with AF in canine lymphoid tumor cells. Therefore, combinatorial therapies with AF might result in enhanced efficacy or the possibility of reduced chemotherapy associated adverse effects if reduced cytotoxic drug dosages could be employed.

## Conclusions

These data suggest that AF can inhibit the growth of canine lymphoid neoplasms and initiate apoptosis in vitro at clinically achievable concentrations, associated with attenuated TrxR activity and elevated ROS generation. In addition, AF synergizes with CCNU or DOX in some cell lines. Hence, AF is a reasonable candidate for clinical investigation in canine lymphoma. To that end, a phase-I clinical trial is underway to determine the maximum tolerated dose, dose-limiting toxicity, and pharmacokinetic/pharmacodynamic parameters of orally administered AF in dogs with spontaneous cancer.

## Abbreviations

AF: Auranofin
CCNU: Lomustine
CI: Combination index
DOX: Doxorubicin
DRI: Dose reduction index
IC50: 50% inhibitory concentration
NHL: Non-Hodgkin lymphoma
ROS: Reactive oxygen species
TrxR: Thioredoxin reductase
